# Treatment of Traumatic Tetanus by Subcutaneous Injection of Carbolic Acid

**Published:** 1890-06-14

**Authors:** 


### TREATMENT OF TRAUMATIC TETANUS BY SUBCUTANEOUS INJECTION OF CARBOLIC ACID.

Drs. Bacelli and Paolini, of Rome, having recently reported successful cases of this mode of treatment, Dr. Bidder, of Berlin, has referred to a case in which he had employed the same fifteen years ago, though he did not publish it at the time. It was that of a child three and a-half years old whose hand was caught by the roller of a chaff-cutting machine which crushed the third phalanges of the third and fourth fingers. The child was intensely dirty. Typical tetanus and trismus set in on the fourteenth day, when Dr. Bidder having thoroughly cleaned the wounds, applied thick dressings saturated with five per cent. solution of carbolic acid and ordered tablespoon doses of a 3½ per cent. solution of chloral (ten grains) several times a day. A slight improvement followed, and the jaws could be parted just enough to admit of his drinking from a cup, but the attempt to swallow the milk brought on severe tetanic spasms. The wounds having still a very unhealthy appearance he injected into the adjacent subcutaneous tissue of each finger "half a syringeful" of a 2 per cent. solution of carbolic acid, continuing the chloral. General and rapid improvement almost immediately showed itself, the spasms decreased in severity and frequency, and in two days the wound was covered with healthy granulations. In all the child had taken ʒiii. of choral, and was sent home in three weeks perfectly well.

